# Integration of Accelerometers and Machine Learning with BIM for Railway Tight- and Wide-Gauge Detection

**DOI:** 10.3390/s25071998

**Published:** 2025-03-22

**Authors:** Jessada Sresakoolchai, Chayutpong Manakul, Ni-Asri Cheputeh

**Affiliations:** 1 Department of Civil and Environmental Engineering, Faculty of Engineering, Prince of Songkla University, Songkhla 90110, Thailand; chayutpong.s@psu.ac.th; 2 Department of Mechanical and Mechatronics Engineering, Faculty of Engineering, Prince of Songkla University, Songkhla 90110, Thailand; niasri.c@psu.ac.th

**Keywords:** tight and wide gauge, accelerometer data, machine learning, building information modeling, railway maintenance, digital asset management

## Abstract

Railway tight and wide gauges are critical factors affecting the safety and reliability of railway systems. Undetected tight and wide gauges can lead to derailments, posing significant risks to operations and passenger safety. This study explores a novel approach to detecting railway tight and wide gauges by integrating accelerometer data, machine-learning techniques, and building information modeling (BIM). Accelerometers installed on axle boxes provide real-time dynamic data, capturing anomalies indicative of tight and wide gauges. These data are processed and analyzed using supervised machine-learning algorithms to classify and predict potential tight- and wide-gauge events. The integration with BIM offers a spatial and temporal framework, enhancing the visualization and contextualization of detected issues. BIM’s capabilities allow for the precise mapping of tight- and wide-gauge locations, streamlining maintenance workflows and resource allocation. Results demonstrate high accuracy in detecting and predicting tight and wide gauges, emphasizing the reliability of machine-learning models when coupled with accelerometer data. This research contributes to railway maintenance practices by providing an automated, data-driven methodology that enhances the proactive identification of tight and wide gauges, reducing the risk of derailments and maintenance costs. Additionally, the integration of machine learning and BIM highlights the potential for comprehensive digital solutions in railway asset management.

## 1. Introduction

**Figure 1:**
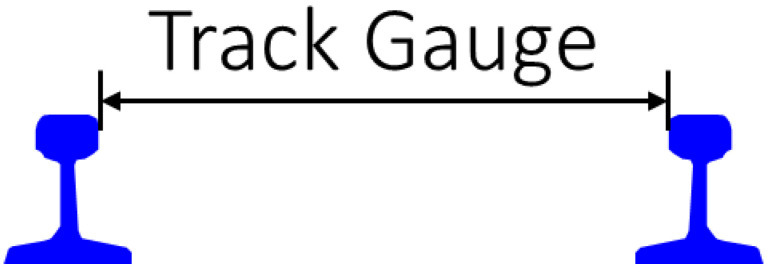
Track gauge measurement.

In the railway system, various parameters influence operational efficiency and safety. One of the most critical factors is track geometry, with gauge being a key parameter. Gauge refers to the distance between the inner faces of two rails, as illustrated in Figure 1. It is a fundamental design and operational aspect that directly impacts the compatibility of rolling stock with track infrastructure, as well as the overall performance, stability, safety, and smooth operation of the railway system. In terms of gauges, significant defects are tight gauges (too narrow) and wide gauges (too wide), which can be caused by wear and tear, thermal expansion, ground settlement, or improper maintenance. These deviations can severely affect train operations and safety because they can lead to derailments, increasing wear, and disruptions to service. Tight gauges can lead to severe hunting, causing trains to have to be stopped sometimes, while wide gauges can lead to a more severe incident, derailment, when the gauge defects are the most common causes of the derailment [1].

According to the report, track gauge defects are responsible for approximately 26 derailments per year, making them the third most common cause of derailments [1]. Therefore, this type of defect is critical, and railway operators have tried to maintain the track gauge according to the intended design to prevent severe incidents.

Track gauge defects can be inspected and measured using different methods, such as visualization, non-metallic tapes, track geometry car [2,3], image processing [4], laser [5], or machine learning [6]. The visualization method is labor-intensive, while other techniques require specialized equipment or tools for measurement. This study aims to utilize acceleration data collected from sensors installed on an axle box, known as axle box accelerations (ABAs), to detect tight and wide-track gauges—an area that has not been previously explored. Additionally, the severity of tight- and wide-gauge conditions will be classified to provide railway operators with the necessary insights for an appropriate response. A benefit of using ABA in this case is that the cost of the installed sensor is relatively cheap compared to other techniques mentioned. Generally, accelerometers have a lower initial installation cost, especially for simple devices, and are easy to install and integrate with existing systems without the need for major infrastructure changes. In contrast, other techniques, such as track geometry measurement, laser scanning, or ultrasonic testing, typically involve higher initial costs due to specialized equipment, dedicated crews, and sometimes additional infrastructure upgrades. When considering operational costs, accelerometers are more cost-effective, as they can continuously collect data without human intervention. They require minimal ongoing maintenance, which mainly involves occasional calibration or sensor replacement. In comparison, other techniques have higher operational costs due to the need for periodic manual inspections or specialized teams to collect data. Some methods, like laser scanning, require expensive equipment and are often more resource-intensive to operate. In terms of maintenance and lifespan, accelerometers tend to be more efficient, the have a long lifespan, and they are relatively low-maintenance, leading to long-term cost savings. They also minimize downtime and do not require track possession time, allowing for continuous monitoring. Other techniques, however, require regular maintenance and recalibration, and they sometimes take the track out of service for data collection, which can lead to operational disruptions and higher costs. While accelerometers provide real-time, continuous data with high accuracy when properly calibrated, other techniques can also offer accurate measurements but often need manual intervention. Some methods may even require track possession, which can cause service disruptions. It is worth noting that the installation position of the accelerometer can significantly affect the quality of the data. Although this aspect is not explicitly addressed in the manuscript, the placement of the accelerometer plays an important role in data accuracy and reliability. In this study, the accelerometers are assumed to be installed on the axle box, which is a standard and commonly used location for capturing axle box acceleration (ABA) data. However, the variations in installation angles or placement can lead to slight differences in the measured accelerations, particularly in the lateral, longitudinal, and vertical directions. To minimize this, the installation position should remain consistent across all measurements. To minimize impacts to the railway operation, effectively implementing real-time data collection using accelerometers on existing rolling stock requires careful planning to minimize operational disruptions. One approach is to install the accelerometers during scheduled maintenance or downtime, ensuring seamless integration into the existing workflow. Additionally, installation can be completed before the rolling stock enters operation, allowing secure sensor mounting and calibration without affecting active service. Using wireless accelerometers can further simplify installation by eliminating the need for extensive wiring. Moreover, IoT-based remote data transmission enables continuous monitoring, reducing the need for manual data retrieval. Proper calibration and testing before deployment are essential to ensure data accuracy and reliability. By strategically timing installations and utilizing advanced data transmission technologies, accelerometer-based data collection can be effectively implemented without interfering with regular railway operations.

To detect track gauge defects and classify their severity, the machine-learning concept is applied in this case. Because ABA is used to detect track gauge defects and categorize their severity, the data are time-series data. Machine learning is a suitable approach as it can identify hidden patterns in time-series data that are difficult for humans to detect. Additionally, building information modeling (BIM) will be integrated with machine learning to enhance railway asset management. Traditionally, BIM is primarily used during the design and construction phases. However, one of its key advantages is its ability to store data related to objects within the BIM model. Applying BIM to the operational and maintenance stages—which constitute the longest phases of a railway project’s life cycle—can provide significant benefits to the industry. As the project progresses, valuable data continuously emerge, further supporting efficient railway management. Therefore, BIM can be used as a platform to manage data, and the data will be beneficial for machine learning, which requires a lot of data for training. Therefore, the integration between machine learning and BIM will maximize the benefits of both for the railway system.

This study provides some novelties to the railway research area by presenting the integration between BIM and machine learning in tight- and wide-gauge detection, which has never been investigated. More information will be discussed in the literature review. In addition, this study employs the ABAs to detect track gauge defects and classify their severity, areas that have never been investigated as well. The expected benefits of this study are that railway operators can improve their systems’ safety by applying the concept proposed in this study to detect and classify track gauge defects, which are the main causes of derailment, as mentioned. As a result, the overall efficiency of railway operations is expected to improve through enhanced safety, reduced maintenance and possession times, and lower maintenance costs, as regular maintenance will require less time and expense. The proposed approach also enables real-time condition monitoring, as data collection is conducted using installed accelerometers that continuously gather data without disrupting regular operations. Lastly, the integration of BIM enhances railway asset management efficiency by facilitating continuous data collection, which can be leveraged to train machine-learning models. Therefore, maintenance and other related decision-making can be performed based on data-driving railway organizations to be data-driven, which is the ultimate goal of different organizations around the world nowadays.

## 2. Literature Review

As mentioned, the track gauge is one of the critical track geometry parameters because it can lead to derailment. Therefore, railway operators have their approaches to managing track gauges in good conditions. The management covers the inspection, measurement, and maintenance. One of the challenges is the detection process of gauge anomalies. The detection can be performed by visualization, non-metallic tapes, track geometry car [2,3], image processing [4], laser [5], or machine learning [6], as mentioned. More details will be presented in this section.

Ghiasi et al. [7] proposed a framework developed by using machine-learning techniques to inspect track geometry using track geometry cars. They applied support vector machine (SVM) to develop machine-learning models. They found that the developed machine-learning model could improve the accuracy by 12% compared to the traditional method. The same finding was found in a study performed by Nugraha et al. [8], who applied machine-learning techniques to detect track anomalies. The technique they used was also SVM, as in the previous study. Other techniques can also be used to detect track anomalies, such as trackside equipment [9], electrical approach [10], mathematical model [11], or image processing [12]. From the literature review, it can be seen that one of the challenges is that the inspection requires some complex methods or equipment to collect data, resulting in additional costs and resource consumption. That is why this study aims to develop a simple approach used to detect tight and wide gauges, and area that has never been investigated.

Although BIM is widely used in the architecture, engineering, and construction (AEC) industry, its application in the railway sector remains limited. The full potential of BIM can be realized when it is utilized throughout the entire life cycle of a project or for asset management. This is because BIM is designed as a data management platform capable of storing comprehensive information within its models. Restricting its use to only the design and construction phases significantly limits its benefits. Previous studies have explored the application of BIM in railway asset management, demonstrating its potential in this field, such as defect detection [13], defect localization [14], or railway maintenance [15]. The previous studies shared the same findings that the application of BIM could improve the overall efficiency of railway asset management in regard to different aspects, such as better collaboration, data integration, and data management. However, as mentioned, the application of BIM in the railway industry is relatively limited compared to the AEC industry. Therefore, this study also aims to develop an approach to integrate BIM and machine learning for defect detection and severity classification, especially track gauge anomalies.

The literature review reveals that the integration of BIM and machine learning has not been explored for detecting track gauge anomalies, specifically tight and wide gauges. This represents a significant research gap that this study aims to address. Additionally, this study investigates more advanced machine-learning techniques beyond those used in previous studies, which primarily relied on support vector machines (SVMs). Unlike past research, this study specifically focuses on tight and wide gauges—an area that has not been previously examined.

The data used in this study consist of axle box accelerations (ABAs), which can be collected during regular operations by installing accelerometers on the axle box of rolling stock. This method is simpler and more cost-effective compared to those used in previous studies. By integrating BIM with machine learning, railway operators can benefit from improved track gauge defect detection and severity classification, enhanced data utilization, real-time condition monitoring through continuous accelerometer-based data collection, better cost management, increased safety, and overall improvements in maintenance efficiency.

## 3. Methodology

This study utilizes numerical models to investigate and analyze the dynamic behavior of rolling stock and track gauge anomalies. The software employed for this analysis is Universal Mechanism (UM) Version 9, a dynamic multibody simulation (MBS) tool. The software’s capabilities vary based on the installed packages. In summary, UM will simulate the dynamic behavior of rolling stock running on tracks with different track gauge anomaly conditions.

Axle box accelerations (ABAs) from three axes—longitudinal (X), lateral (Y), and vertical (Z)—will be exported as time-series data and stacked into a three-layer one-dimensional format. These processed data will then be used to train machine-learning models for defect detection and categorization. Finally, the machine-learning predictions will be integrated with the developed BIM model to provide additional benefits in railway asset management. Further details will be provided in the following sections.

### 3.1. Numerical Model Development and Validation

In this study, the MBS model is developed based on the Manchester benchmark rolling stock. The original model was developed in 1998, allowing researchers to use this benchmark to compare results from different software [16]. Different results from various software and simulations were published in the mentioned reference. Examples of software are ADAMS/Rail, MEDYNA, GENSYS, NUCARS, SIMPACK, and VAMPIRE.

**Figure 2:**
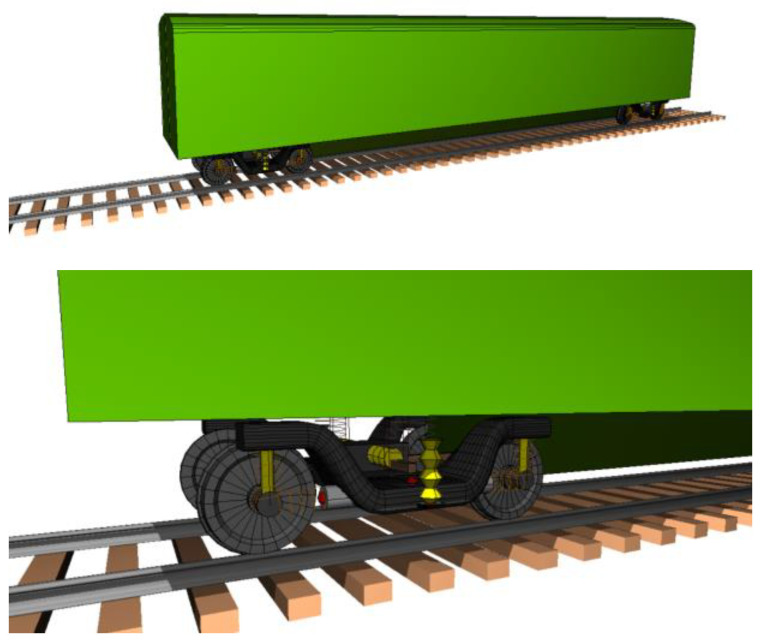
Examples of MBS model based on Manchester benchmark.

The MBS model is developed based on the subsystem technique. The components of the rolling stock consist of two bogies, and each bogie is supported by two wheelsets. Each wheelset is supported by the primary suspensions, which have longitudinal, lateral, and vertical stiffnesses. The dampers have the function of dissipating the vertical accelerations only. Each wheelset also contains the secondary suspensions containing the vertical stiffnesses. Different from the primary suspensions, the secondary suspensions have dampers in both vertical and lateral directions. Additional components of the secondary suspensions are the roll bar, which is used to reduce the body roll of rolling stocks; and a pair of lateral bump stops to be the cushions or pads, limiting the movement of the secondary suspensions. Examples of the MBS model are shown in Figure 2. One of the advantages of UM is that the processing time of UM is faster than that of other software [17].

To validate the developed MBS models, the Manchester benchmark [16] is referred to. Different parameters can be used to compare. Different resulting parameters were presented in the said references. Examples of benchmark parameters are natural frequencies of sway, yaw, bounce, pitch, and longitudinal directions; Eigenvalues of the mentioned movements; lateral wheelset displacements; total forces of each wheel; contact angles of each contact point; and creepages; creep forces, and normal forces of each contact point. In this case, ABAs are the main parameters used to detect and classify tight and wide gauges. Therefore, the parameters selected to validate the MBS model should be able to significantly represent ABA. Considering this issue, lateral wheelset displacements and the total forces of each wheel are used to validate the MBS model. The results from ADAMS/Rail are used to compare and validate the MBS model. The comparisons are shown in Table 1.

From Table 1, it can be seen that the results from UM and ADAMS/Rail are close in most cases. Most of the compared parameters have differences of less than 10%. For the compared parameters with big differences, their scales are small, so the small values result in relatively big differences. However, the average difference between the results from UM and ADAMS/Rail is about 8.4%, which is less than 10%. Therefore, it can be concluded that the MBS model developed using UM is reliable and can be used to represent the dynamic behavior and interaction between rolling stocks and tracks. In this case, UM will be used to model the dynamic behavior of ABAs under tight- and wide-gauge conditions.

### 3.2. Data Variation and Characteristics

In this study, data were numerically simulated by using validated MBS models, as mentioned in the previous section. The software used was UM. To develop machine-learning models and mimic the real dynamic characteristics of tight and wide gauges, data variation is crucial to create data diversity and ensure that the developed machine-learning model will be comprehensively able to be applied in various situations. Different parameters are varied to simulate the MBS models. Varied parameters consist of speeds of rolling stocks, weights of rolling stocks, and sizes of track gauge anomalies (tight and wide gauges). The summarized data variation is shown in Table 2. From data variation, the total number of samples for training the machine-learning model is 1309 samples. Data splitting is conducted using stratified sampling. Samples are split into two subsets, with 70% allocated for training the model and 30% reserved for testing its performance. When the length of the track section is 500 m. The data variation shown in the table is ranged based on related standards and use cases in real life. For the speeds of rolling stocks, this range of speeds should be comprehensive for most of the use cases in reality, as well as the weights of rolling stocks. For the size of track gauge anomalies, the numbers are defined based on the standard of NSW Transport Rail Corporation in Australia [2], outlining that the wide gauge should be smaller than 21 mm, while the tight gauge should be smaller than 10 mm; otherwise, maintenance is required to be performed. From the referred standard, the urgency of the response is categorized into six classes, as shown in Table 3. Because this study aims to develop the approach to detect tight and wide gauges in the early stage and avoid unexpected failure and maintenance, the targeted response category is normal.

In addition, the response category also depends on the track speed, which is shown in Table 4. From the table, it can be seen that the tracks with higher speeds will be prioritized. In this study, the maximum speed of rolling stocks is 250 km/h, which is higher than 160 km/h. Therefore, to maintain the response category as normal, the thresholds of tight and wide gauges are less than 10 and 21 mm, respectively. Therefore, this study defined the range of tight and wide gauges within this range.

**Figure 3:**
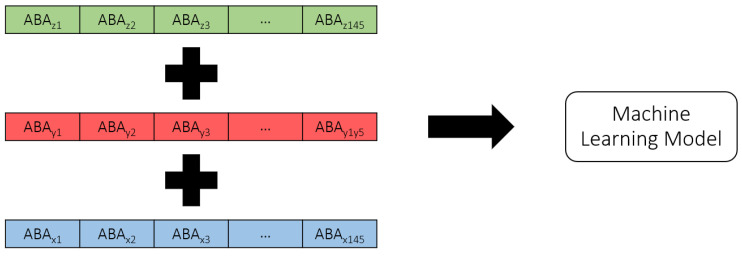
Shape of outputs from the MBS models or the features for the machine-learning model.

UM is flexible in different aspects. One of the flexibilities is exported data or results. Therefore, there are multiple options for selecting MBS models’ output to be used as features for machine-learning model training. In this case, as mentioned, ABAs will be used as features to train the machine-learning model. However, multiple options are still available, such as different axes of ABAs, as well as their numbers. In this study, ABAs from three axes, longitudinal (X), lateral (Y), and vertical (Z) directions, are used to train the machine-learning model. Each ABA will be in the form of one-dimension time-series data. Each of them will be stacked with each other to be fed into the machine-learning model. The shape of outputs from the MBS models or the features of the machine-learning model are shown in Figure 3. From the figure, the shape of each ABA is [1 × 145] when 145 is the number of timesteps or data points exported from the MBS model. This number can be varied based on the speeds of rolling stocks and the frequency of calculation. However, from the preliminary investigation, the author found that this frequency number is optimal in terms of processing time and model reliability.

**Figure 4:**
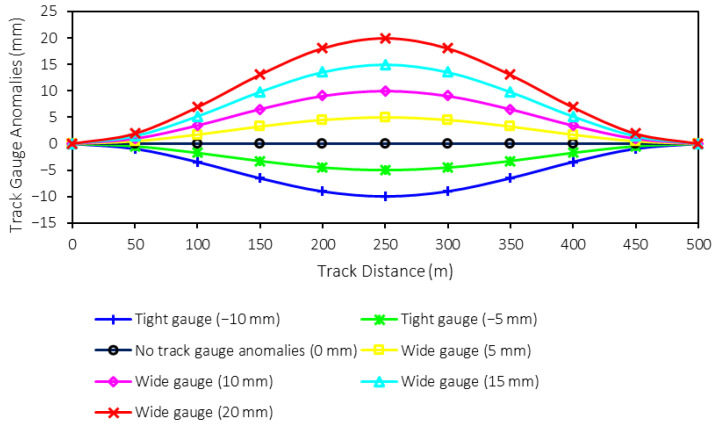
Shapes of track gauge anomalies.

For the track gauge anomalies, namely tight and wide gauges, the sizes are varied, as mentioned in Table 2. From the table, the range of track gauge anomalies is −10 (tight gauge) to 20 (wide gauge) mm when the step of variation is 5 mm. Therefore, there are six sizes of trach gauge anomalies plus one perfect track condition, which can be seen in Figure 4. These different sizes of track gauge anomalies will also be used as classes or labels for machine-learning model prediction. Therefore, there are seven classes (0–6) for the prediction. It is worth noting that one rail is assumed to be in perfect condition when the other rail has the shape deformation to create track gauge anomalies.

**Figure 5:**
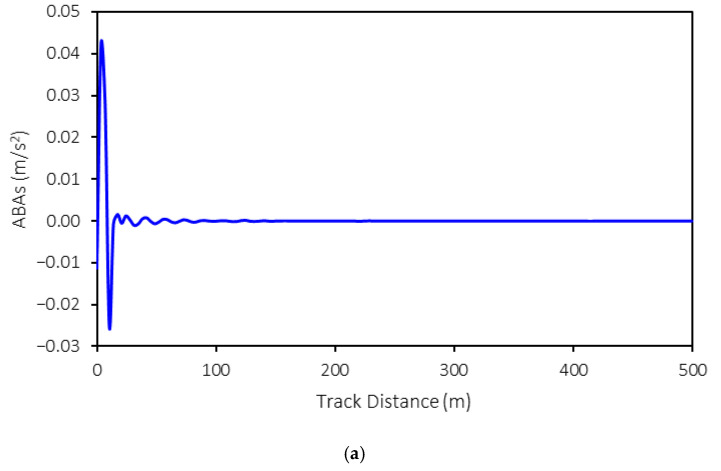

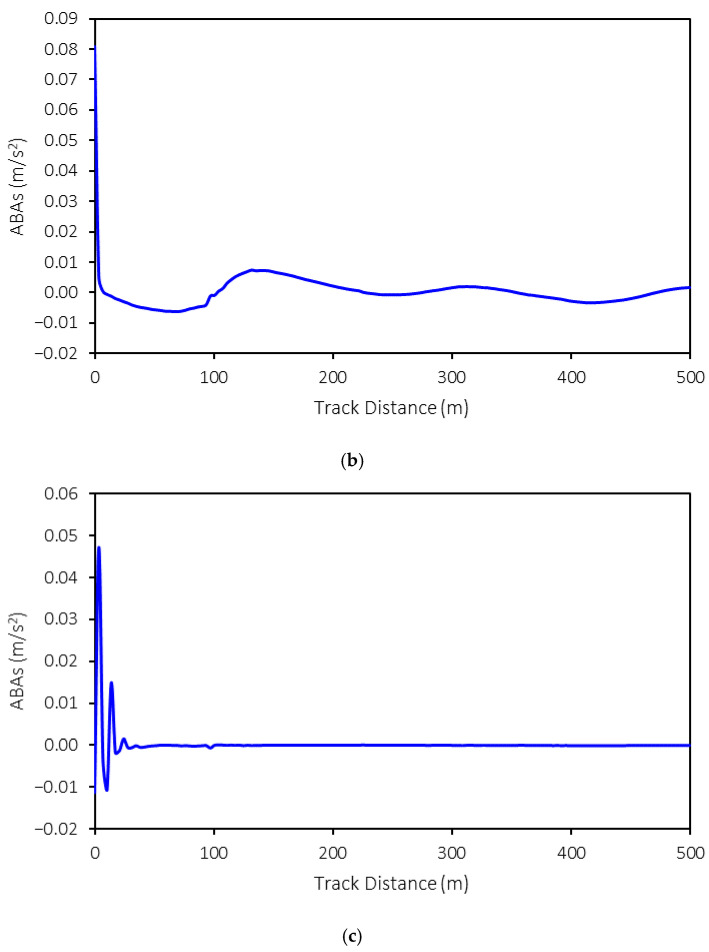
Examples of exported ABAs: (**a**) X-longitudinal direction, (**b**) Y-lateral direction, and (**c**) Z-vertical direction.

The three-dimensional axle box accelerations (ABAs) from the MBS models are exported for further development of the machine-learning model. An example of ABA is shown in Figure 5. In this example, the following conditions are presented: a rolling stock speed of 50 km/h, a weight of 15 tons, and tight-gauge anomalies with a 10 mm variation. As shown in the figure, the ABAs in the longitudinal and vertical directions exhibit relatively large magnitudes at the start of the simulation that then decrease as the simulation progresses. This is because no irregularities are present in these directions. However, the ABAs in the lateral direction vary throughout the simulation, reflecting the impact of tight and wide gauges on the rail profile in this direction. Consequently, the ABAs in the lateral direction fluctuate throughout the simulation.

### 3.3. Machine-Learning Model Development (Data Processing, Data Format, and Sample Size)

**Figure 6:**
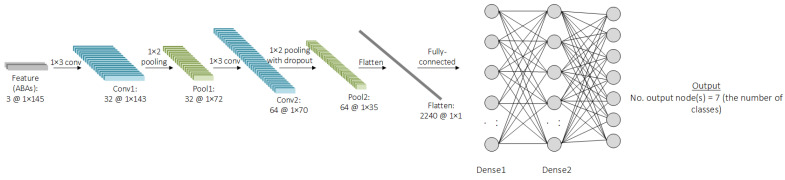
Example of the CNN architecture.

To develop the machine-learning model, different machine-learning algorithms that are suitable for time-series data are preliminarily investigated, such as convolution neural network (CNN), long short-term memory (LSTM), recurrent neural network (RNN), and ResNet. It is found that CNN tends to provide the best accuracy with the shortest training time. It is worth noting that other traditional machine-learning algorithms, such as artificial neural network (ANN), multiple regression (MR), decision tree (DT), random forest (RF), or support vector machine (SVM), are not appropriate for time-series data due to their limitations in architectures. While the literature may not definitively recommend a superior model for time-series data, this study undertook a thorough exploration and evaluation of various machine-learning models that are well-regarded for their performance with time-series data. Given the specific requirements of this study—namely, the detection and classification of track gauge anomalies from ABA data–several models have been tested to determine the most effective approach. Through a comprehensive analysis, it became clear that CNN outperformed the other models in terms of both accuracy and training time. This performance advantage made CNN the optimal choice for this study’s objectives. While this result demonstrates the model’s suitability in this context, it is important to note that, for different applications or datasets, railway operators or responsible parties should consider testing various machine-learning models to determine the most appropriate algorithm for their specific needs. Therefore, CNN will be further investigated in this study. The problem in this study can be defined as a classification study because the labels are discrete. To preprocess the data, the raw axle box acceleration (ABA) data collected from accelerometers installed on the axle boxes of rolling stock are utilized. Before the ABA data can be used for machine-learning model training, several preprocessing steps are undertaken. These steps include rearranging the raw data into a structured format, normalizing the values to ensure consistency, and reshaping the data to address any inconsistencies in its shape. In particular, when data are collected at a fixed frequency while the rolling stock speeds vary, the length of the track sections can remain constant, but the shape of the ABA data will vary. Such variation makes the data unsuitable for direct machine-learning model training. To resolve this, the data are reshaped. One technique employed to achieve consistent data shapes is padding samples with zeros in cases where the data shape is smaller than others. For example, samples from rolling stock running at higher speeds generate shorter data. By adding zeros to these samples, the shape is standardized, making the data suitable for training the machine-learning model. Furthermore, the 1D time-series ABA data from the three axes (longitudinal, lateral, and vertical) are stacked to form a three-layer 1D time-series dataset, enabling the model to learn from multi-dimensional inputs. The processed data are then saved in CSV format for efficient loading and training, as shown in Figure 3. Then, the shape of the feature will be changed, along with the process, inside the CNN model. This will depend on the architecture of the CNN model, such as the number of filters, the number of kernels, the number of convolutional layers, the number of pooling layers, or the pool size. The CNN is a type of deep-learning model designed to process structured data, like images, videos, or time-series data. It works by automatically extracting and learning features from the features through a series of interconnected layers. The features are passed through convolutional layers, where filters and kernels slide over the data, performing element-wise multiplications to create feature maps that highlight specific patterns. After each convolutional operation, an activation function like the rectified linear activation function (ReLU) introduces non-linearity, enabling the model to learn complex hidden patterns. To reduce the spatial dimensions and computational complexity, pooling layers, such as max pooling or average pooling, are used to retain the most important features while discarding unnecessary details. The feature maps are then flattened into a one-dimensional vector and passed to fully connected layers, which act as a classifier. These layers combine the extracted features to produce the final predictions. The entire network is optimized by minimizing a loss function, such as cross-entropy for classification tasks, using optimization algorithms like Stochastic Gradient Descent (SGD) or Adam. CNNs are highly efficient at automatically extracting hierarchical features and are widely used in tasks such as image recognition, object detection, video analysis, and time-series analysis due to their ability to generalize well on large datasets. An example of the CNN architecture is shown in Figure 6. From the figure, it can be seen that the feature-extraction part contains different CNN components and is connected with the classification part consisting of dense layers, similar to the ANN model. The number of nodes in the input layer is equal to the number of features flattened from the feature-extraction part when the number of nodes in hidden layers can be varied. Last, the number of nodes in the output layer is equal to the number of classes in the case of the classification problem.

### 3.4. Hyperparameter Tuning

Hyperparameter tuning is the process of optimizing the external configurations of a machine-learning model that are not learned during training but are set beforehand. These hyperparameters include the learning rate, batch size, number of layers, filter sizes, and dropout rate. Hyperparameter tuning plays a crucial role in determining the model’s performance and its ability to generalize to unseen data. Effective tuning ensures that the model strikes a balance between underfitting and overfitting, thereby improving its accuracy and robustness. Some common methods for hyperparameter tuning are manual search, grid search, and random search. Manual search relies on trial and error, guided by intuition or domain knowledge, but can be time-consuming. Grid search exhaustively evaluates all possible combinations of hyperparameters within specified ranges; however, it becomes computationally expensive for large models. Random search, on the other hand, randomly samples hyperparameters within defined bounds and is more efficient in high-dimensional spaces.

Despite its importance, hyperparameter tuning poses several challenges. It can be computationally intensive, especially for deep models or large datasets. Additionally, the interdependence of hyperparameters complicates the search process, and excessive tuning risks overfitting the validation set. To mitigate these issues, it is advisable to start with a smaller subset of the data for quicker iterations, monitor metrics such as validation loss or accuracy to assess progress, and adopt efficient techniques like random search or grid search. With these strategies, hyperparameter tuning can significantly enhance the performance and reliability of machine-learning models. In this study, grid search is employed because it is better suited for situations where the hyperparameter space is defined and all combinations are predictable. Grid search ensures that all combinations are explored thoroughly, providing confidence that the selected configuration is optimal within the defined range. In addition, this technique is reliable and comprehensive. The list of tuned hyperparameters of the CNN model is shown in Table 5. To evaluate the performance of the developed machine-learning model, indicators related to the classification are used. In this study, precision, recall, F1-score, and accuracy are used. The calculation of each is shown in Equations (1)–(4), where true positive (TP) is the correct prediction of positive cases, true negative (TN) is the correct prediction of negative cases, false positive (FP) is the incorrect prediction of positive cases (actual negatives), and false negative (FN) is the incorrect prediction of negative cases (actual positives). For definitions, precision is the proportion of correctly predicted positive cases out of all cases predicted as positive. Recall is the proportion of correctly predicted positive cases out of all actual positive cases. F1-score is the harmonic mean of precision and recall, providing a balanced measure of both metrics, especially useful when the dataset is imbalanced. Last, accuracy is the proportion of correctly predicted cases (both positive and negative) out of all cases.

The preferable values for precision, recall, F1-score, and accuracy are close to 1. For the interpretation, precision is crucial where false positives carry a high cost. On the other hand, recall becomes a priority when false negatives are more critical, as missing actual positive cases can have severe repercussions, thus making recall suitable for this study because it aims to avoid undetected anomalies. The F1-score provides a balanced measure of precision and recall and is particularly useful for imbalanced datasets where one class significantly outweighs the other. A high F1-score indicates a good trade-off between false positives and false negatives, making it ideal when both types of errors are important but need to be balanced. Accuracy is straightforward and commonly used because it is most meaningful. Therefore, accuracy can reliably indicate the model’s performance. From their definitions, this study will consider all indicators to evaluate the model’s performance.

(1) $$Precision=\frac{TP}{TP+FP}$$

(2) $$Recall=\frac{TP}{TP+FN}$$

(3) $$F1-score=2\frac{Precision*Recall}{Precision+Recall}$$

(4) $$Accuracy=\frac{TP+TN}{TP+TN+FP+FN}$$

### 3.5. BIM Model Development

To develop the BIM model for the railway project, an approach presented in [14] is employed. The software used to develop the BIM model is AutoCAD Civil3D 2024, which has the potential to develop BIM models to achieve the nD BIM model. In this study, the BIM model is aimed to be 6D when the 4D, 5D, and 6D are time, cost, and maintenance aspects, respectively. The BIM model can be developed using the following steps.

First, the simplest BIM model is developed to be a 3D BIM model, which is a common BIM model in the AEC industry. However, BIM models in the railway industry have different sequences to develop. The 3D BIM model is developed based on vertical and horizontal alignments. Other parameters are also crucial for the correction of the models, such as gradients, radiuses of curvature, and superelevation. The model’s level of detail is defined based on sample lines. The smaller sample lines refer to the more detail of the models. When alignments in both directions are modeled, other components can be placed along the alignments, such as rails, sleepers, rail pads, track structures, or retaining walls. These elements are modeled as 3D solids, which can be further exported as Industry Foundation Classes (IFCs), the industrial standard format of BIM models.

Then, the 3D BIM models are further developed to achieve 4D by linking each element to a scheduling software, such as Navisworks, Microsoft Project, or Primavera P6, to create project schedules. Then, the 5D BIM models can be simply achieved because 3D solid elements have volume properties that can be used to calculate quantities and costs. Last, the 6D BIM models aim to fulfill the requirement of maintenance aspects and other asset management purposes. Additional property sets can be added to BIM objects or 3D solids in this case. The additional property sets can be any parameters defined to fulfill the maintenance aspects, such as defects and maintenance requirements. Until this stage, the developed BIM model is 6D and is ready to be integrated with machine-learning models.

### 3.6. Machine Learning and BIM Integration

**Figure 7:**
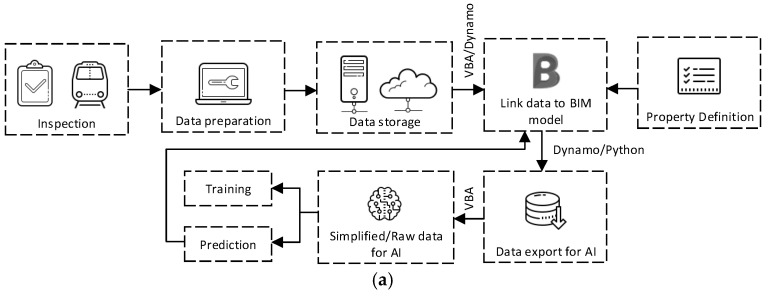

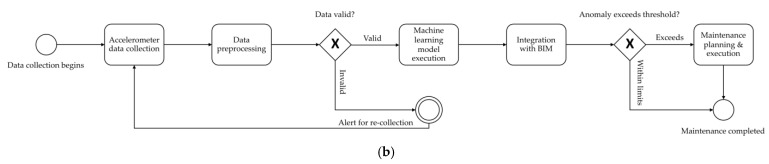
Workflow of the developed approach (**a**) for integrating machine learning and BIM, and (**b**) BPMN (Business Process Model and Notation).

To integrate machine learning and BIM, the workflow is developed based on the workflow presented in [14]. The developed workflow is shown in Figure 7a. From the figure, the inspection data or ABAs are collected from the installed accelerometer. Then, raw data are processed to be in the form that is appropriate for being stored in the BIM models and fed into the machine-learning model. To store the processed data in the BIM model via Property Definition, Autodesk Dynamo, which is a visual programming environment, and with Visual Basic for Applications (VBA) are used. Data related to each element of the 3D solid can be linked and stored in the BIM model in this step. Now, the developed BIM model is a data-rich model containing plenty of data. To exchange and utilize stored data with machine-learning models, Dynamo and Python scripts are used to manage and exchange stored data with spreadsheet software or machine-learning models. Then, data are ready to be used to train machine-learning models or for other artificial intelligence (AI) purposes. In the case of the machine-learning model testing or model employment, the predictions or outputs from the machine-learning models can be stored in the BIM model by using Dynamo and Python script 3.7.0, just as when they are initially exchanged with the machine-learning models. It is worth noting that Dynamo is not a ready-to-use software solution but rather a platform that provides a flexible environment for developing customized workflows for BIM element management. In this study, Dynamo is used to build a tailored workflow specifically designed for the data exchange between BIM and the machine-learning model. While the platform offers a range of functions, it requires the development of a custom workflow to address the unique needs of this study, such as integrating track gauge anomaly data with machine-learning predictions. The aim of this study is to propose a methodology for utilizing Dynamo to create this custom workflow, enabling efficient data exchange and model integration. Now, the integration between machine learning and BIM is complete and seamless, which will be beneficial for railway operators for railway asset management.

For the data flow between BIM and the machine-learning model, the components in the BIM model must first be designed through property definition at the outset of the BIM model development. This step ensures that railway operators or responsible personnel can store the necessary information within the BIM model. In this case, examples of stored information include track gauge anomalies and maintenance requirements. During the property definition phase, different data formats can be defined: for instance, track gauge anomalies are stored as real numbers, while maintenance requirements are stored as text. When information is exported or exchanged between the BIM model and the machine-learning model, the type of data is identified by its name. As illustrated in the figure, data exchange is facilitated using Dynamo, which offers ready-to-use functions or can be customized through VBA or Python. In this case, the information stored in the BIM model can be exported as a spreadsheet or CSV file using Python. The exported file will contain data such as track component IDs, their locations, track gauge anomaly values, and maintenance requirements. Once predictions from the developed machine-learning model are available, Python can be used to merge these predictions with the exported spreadsheet file from the BIM model. This file, which contains the defined information, is then imported back into the BIM model using Python or VBA code. This process of data exchange can be carried out regularly whenever new data are available, the machine-learning model is retrained, or new predictions are generated.

In this study, a Business Process Model and Notation (BPMN) is performed to visualize the data flow between BIM and the machine-learning model. BPMN is a standardized graphical notation that is commonly used to model business processes and workflows. This methodology is applied with the aim of providing a clearer, more structured, and standardized representation of the interactions and processes involved in the integration of BIM and machine learning. The BPMN diagram effectively depicts the sequence of activities, decision points, and data exchanges that occur between these two components, as is essential for understanding how data are processed and utilized by the machine-learning model. This approach enhances the clarity and comprehensibility of the workflow, allowing readers to easily follow the complex interactions between BIM and machine learning. The use of BPMN ensures that the model’s architecture is presented in a standardized manner, facilitating communication across disciplines and ensuring that both technical and non-technical audiences can better grasp the integration of these technologies. The BPMN diagram is shown in Figure 7b. The process initiates with the systematic collection of accelerometer data, a critical step that captures dynamic measurements of acceleration. These measurements serve as the foundational dataset for subsequent analytical procedures. Following data acquisition, the raw accelerometer data undergo a comprehensive preprocessing phase. This phase is essential to ensure data integrity and suitability for machine-learning model development. Preprocessing typically involves a series of steps, including data cleaning, reshaping, and transformation to convert the data into a format amenable to machine-learning algorithms. Once preprocessed, the data are utilized as input for a machine-learning model. The execution of this model involves the application of sophisticated algorithms designed to identify patterns, correlations, and predictive insights within the dataset. The outcomes generated by the machine-learning model are then integrated with BIM. This integration facilitates a holistic view, enabling the correlation of predictive insights with spatial and structural data. The final phase of the process focuses on maintenance planning and execution. Leveraging the integrated data, maintenance activities are strategically planned to optimize resource allocation, enhance operational efficiency, and preemptively address potential issues. This approach not only supports proactive maintenance strategies but also contributes to the longevity and sustainability of the infrastructure. By combining machine learning with BIM, the process offers a comprehensive framework for informed decision-making and efficient maintenance management.

## 4. Results and Discussion

### 4.1. BIM Model Development Outcome

**Figure 8:**
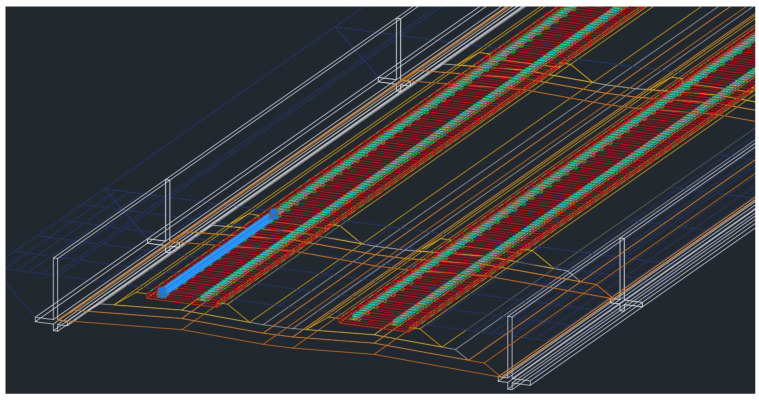
Developed a BIM model for the railway project.

**Figure 9:**
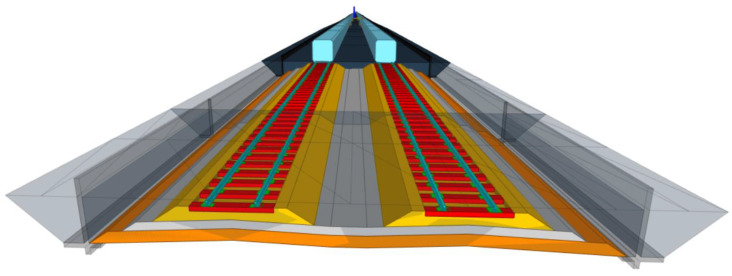
Exported BIM model as an IFC file.

From the BIM model development sequences presented in the previous section, the outcome is shown in Figure 8. From the figure, different track elements are included in the model, such as rolling stock envelop demonstrating the clearance, rails, rail pads, sleepers, track structures, and retaining walls. Other elements can also be included in the BIM model according to the defined level of detail. These elements are modeled as 3D solid objects in the BIM model, and 4D to 6D can be achieved using the described approach in the previous section. Then, the developed BIM model can be exported as an IFC file, which is the standard format for BIM. The result from the exported BIM model as the IFC file is shown in Figure 9.

**Figure 10:**
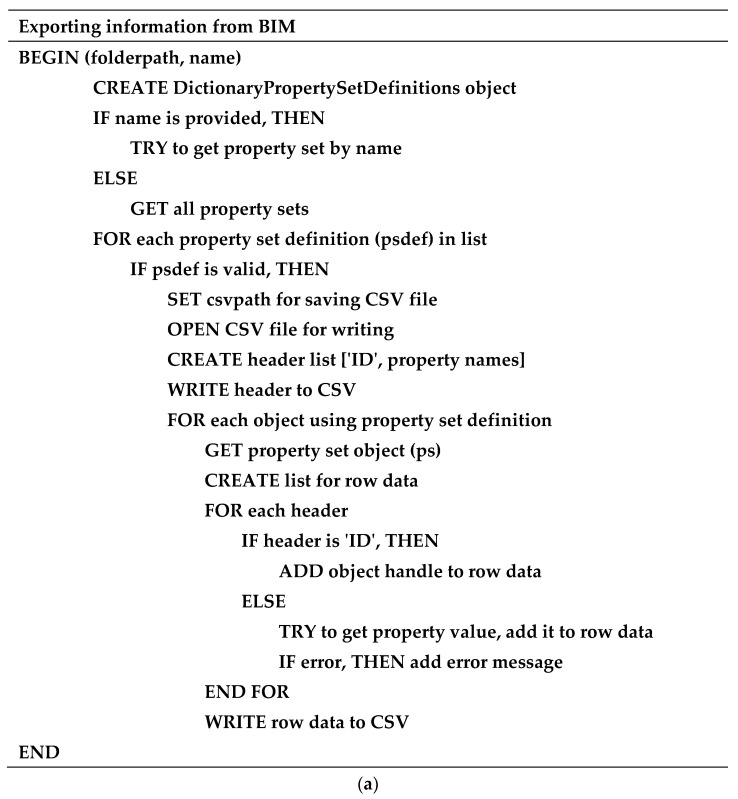

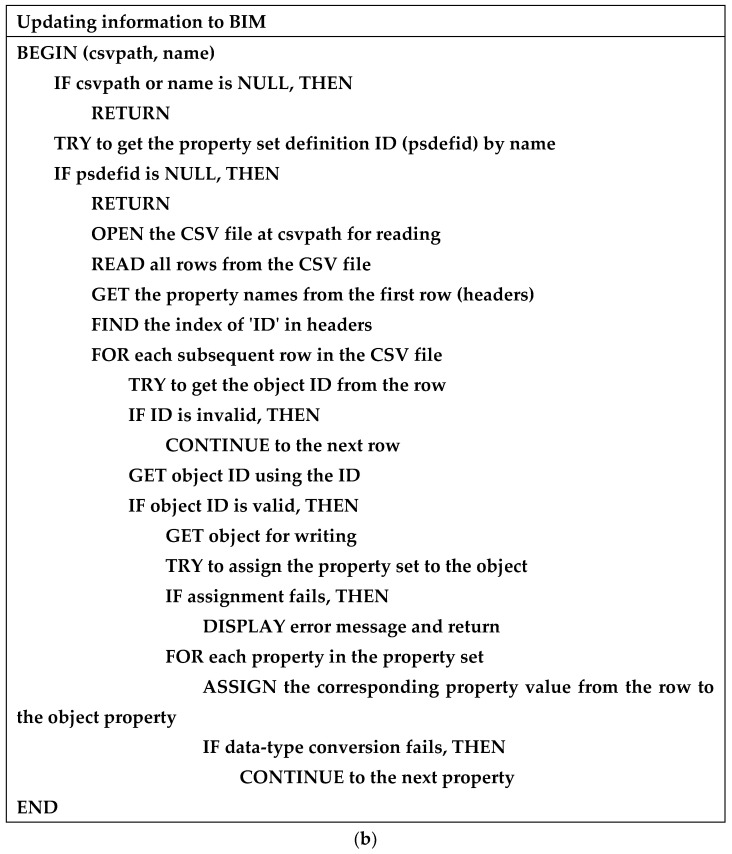
Examples of pseudo-code for (**a**) exporting information from the BIM model and (**b**) updating the information in the BIM model.

The data that are designated to be stored in the BIM model can be conducted through property set definitions, as mentioned in the previous section. The property set definitions can be selected to include in any 3D solid objects which are compatible with the developed BIM model and the proposed approach. In this study, the sixth dimension of the BIM model is about the maintenance aspects. Therefore, they are defined as emerging defects and maintenance requirements. In this case, the focused defects are track gauge anomalies consisting of tight and wide gauges. At the same time, the maintenance requirement is the need for track gauge correction. This information can be determined based on the prediction from the machine-learning model, which will be presented in this section. Then, the predicted defects and maintenance requirements will be included in the BIM model using the help of Dynamo and the Python scripts, as mentioned. To improve the clarity and reproducibility of the integration process between BIM and machine-learning models, pseudo-code is performed. The pseudo-code offers a structured, language-agnostic representation of the workflow, illustrating key integration steps. By using pseudo-code, a clear step-by-step understanding of the process is provided, which enhances transparency and facilitates the practical application of the developed approach. This addition will be valuable for researchers and practitioners seeking to replicate or build upon the work. Examples of the pseudo-code for exporting the information from the BIM model and for updating the information in the BIM model are shown in Figure 10.

**Figure 11:**
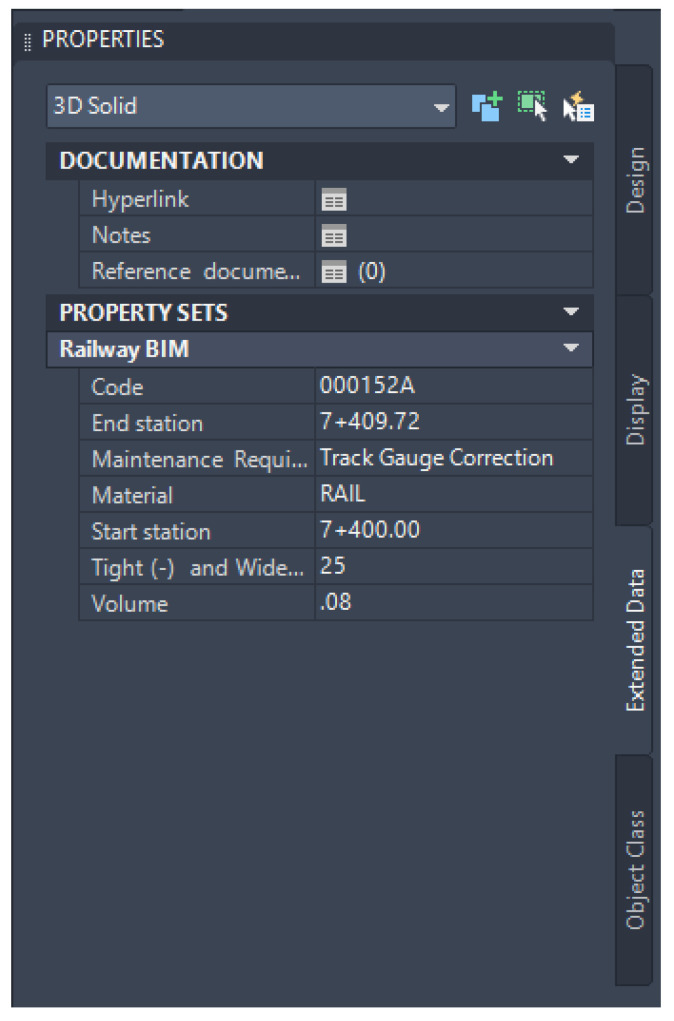
Integrated information in the BIM model for the maintenance aspects.

This integration will be beneficial for railway operators in enhancing efficiency, optimizing asset management, and improving overall operational performance. This also supports predictive maintenance by identifying when track gauge anomalies are likely to emerge based on real-time data, reducing unplanned downtime and maintenance costs. The outcomes from the data integration from the machine-learning model to the BIM model can be shown in Figure 11. From the figure, the size of tight and wide gauges is included. The maintenance requirement will be determined based on the size of the track gauge anomalies shown in Table 2. If the number is larger than the defined thresholds, the maintenance requirement will automatically identify as track gauge correction required. The safety of the system also benefits from this integration, as machine learning can be used to identify the possibilities of emerging defects. BIM can be used to visualize areas with high possibilities of defects and acknowledge responsible people. The integration of BIM and machine learning will support the decision-making in railway asset management. In addition, the integration also supports maintenance cost reduction and sustainability due to better maintenance efficiency.

### 4.2. Machine-Learning Model Development

From the machine-learning model development, different combinations of hyperparameters via hyperparameter tuning, and the optimal hyperparameters are shown in Table 6.

**Figure 12:**
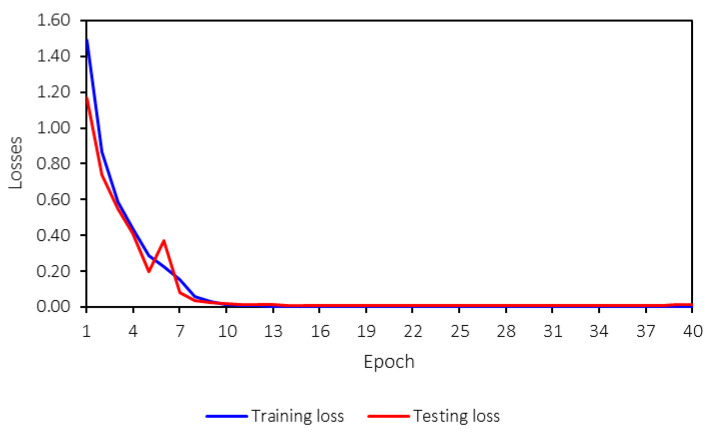
Training and testing losses.

The machine-learning model is trained for 40 epochs. While this number may seem low, the training converged effectively within this range, as indicated by the training process. Figure 12 shows the loss values for both training and testing, and it is evident that the model converges after 10 epochs. Therefore, 40 epochs are sufficient for training the model in this case. The machine-learning model reaches saturation before 10 epochs, with loss and accuracy values stabilizing through the 40th epoch. This early convergence indicates that the model has already achieved optimal performance within the initial training phase. The training is extended to 40 epochs to ensure a comprehensive evaluation and confirm that no further improvements occur beyond convergence. Additionally, the training time does not differ significantly between 10 and 40 epochs, allowing for extended training without compromising efficiency. This stability further suggests the model’s robustness, as both training and testing losses and accuracies remain consistent throughout the process.

**Figure 13:**
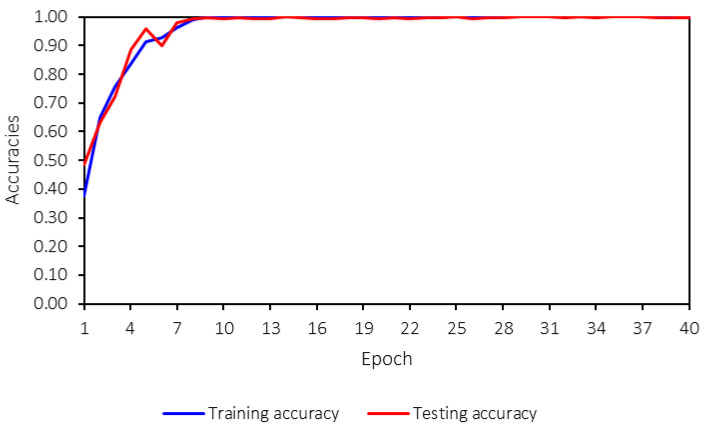
Training and testing accuracies.

Both the training and testing losses follow the same trend and remain close to each other. A key concern in machine-learning model training is overfitting, which can be identified by several signs, such as significant differences between training and testing losses, high variance in the testing loss during training, declining accuracy, or large discrepancies between training and testing accuracies. Figure 13 presents the training and testing accuracies.

From Figure 12 and Figure 13, no overfitting is observed, as both accuracies and losses for training and testing are consistent and follow the same trend. Furthermore, the trend is strong, stable, and converges to the desired values—0 for losses and 1 for accuracies. This indicates that the developed machine-learning model performs well and that hyperparameter tuning has been appropriately applied.

Besides comparing training and testing accuracy, to verify the overfitting, cross-validation is also conducted to ensure that the trained model does not have the overfitting issue. In this study, 5-fold cross-validation (k = 5) is performed. Cross-validation is a widely used technique for assessing model performance by partitioning the dataset into multiple subsets, where the model is trained on a portion of the data and tested on the remaining portion in an iterative manner. This approach helps mitigate the risk of overfitting by ensuring that the model is evaluated on different segments of the dataset, reducing dependence on a single training–test split. The results of the cross-validation process, including the mean and standard deviation of accuracy, provide insights into the model’s stability across different data splits. By incorporating this validation strategy, we further demonstrate that the high accuracy reported is not due to data leakage or an overly specific training dataset but rather reflects the model’s true predictive capability across varying track gauge conditions. In addition, to demonstrate the performance of the model based on the number of epochs, the results are shown in Table 7.

The results from the cross-validation provide insights into the model’s generalization ability and potential overfitting concerns. At 10 epochs, the model achieves high accuracy on both the training and testing datasets, with most folds reporting values above 0.98, except for fold 4, which shows a noticeable drop in test accuracy (0.86). This suggests some variability in performance, indicating that the model is still in the early stages of training and has not fully stabilized. As the number of training epochs increases to 20, the training accuracy reaches 1.00 across all folds, and the testing accuracy remains consistently high at 0.99. This trend continues for 30 and 40 epochs, where the model maintains perfect accuracy on the training data (1.00) and near-perfect accuracy on the test data (mostly 0.99, with minor variations down to 0.98 in a few cases). While the consistency in training and testing accuracy suggests that the model is not suffering from significant overfitting since the test accuracy remains high and does not sharply diverge from the training accuracy.

In terms of model performance indicators, precision, recall, F1-score, and accuracy are employed, as mentioned. The calculations are shown in Equations (1)–(4). Unlike accuracy, other performance indicators, such as precision, recall, and F1-score, are calculated separately for each class, meaning that each track gauge anomaly type has its own evaluation metrics. In contrast, accuracy is computed based on the overall prediction performance across all classes. The model’s performance can be assessed using these indicators, as presented in Table 8.

The model is designed to classify seven different types of track gauge anomalies, as outlined in Table 2. The results demonstrate that the model achieves high and satisfactory performance across all evaluation metrics. Specifically, the training accuracy is 1.00, and the testing accuracy is 0.99, indicating a strong generalization capability. Additionally, the precision, recall, and F1-score values are either one or very close to one, further confirming the robustness and reliability of the developed machine-learning model.

These high-performance metrics suggest that the model can accurately detect and classify various track gauge anomalies, making it a valuable tool for predictive maintenance in railway systems. The consistently high values across different classes also indicate that the model does not suffer from significant class imbalance issues and can effectively distinguish between different types of anomalies.

For more detail, the confusion matrix is presented in Table 9. The confusion matrix is a table used to evaluate the performance of a classification model by comparing the predicted and actual classes. It provides a detailed breakdown of correct and incorrect predictions for each class, making it a valuable tool for performance analysis. Rows of the confusion matrix represent the predicted classes, while columns represent the actual classes. Each cell in the confusion matrix presents the number of samples where a specific prediction matches or differs from the actual class. The confusion matrix offers several key benefits when evaluating classification models. First, it provides a detailed breakdown of performance by showing the number of correct and incorrect predictions for each class. This granular insight helps identify specific areas where the model performs well and where it struggles, making it easier to understand the model’s strengths and weaknesses. Additionally, the confusion matrix calculates crucial performance metrics like precision, recall, F1-score, and accuracy, possibly enabling a more comprehensive evaluation. This is particularly important for imbalanced datasets, where accuracy alone may be misleading, as it does not account for the distribution of classes. In addition, the confusion matrix facilitates class-specific error analysis by highlighting false positives and false negatives, helping responsible persons identify patterns in misclassifications. This information can guide improvements, such as model tuning, collecting more representative data, or adjusting class distribution in the model. It is also highly beneficial for each class performance evaluation.

It should be noted that the installation position of the accelerometer can significantly affect the quality of the data. Although this aspect is not explicitly discussed in this study, the placement of the accelerometer plays a critical role in ensuring data accuracy and reliability. In this study, the accelerometers are assumed to be installed on the axle box, which is a standard and commonly used location for capturing ABA data. However, variations in installation angles or placement can lead to slight differences in the measured accelerations, particularly in the lateral, longitudinal, and vertical directions. To minimize these variations, it is important to maintain consistency in the installation position across all measurements. Future research can provide a more detailed analysis of how different installation positions may influence the recorded data and the performance of the machine-learning model, offering valuable insights into optimizing sensor placement for improved data quality.

## 5. Use Case in Practice

This study presents a practical application of the developed approach designed to enhance the monitoring, detection, classification, and maintenance of railway infrastructure by addressing critical issues related to tight- and wide-gauge anomalies. The proposed system leverages accelerometer data, machine-learning algorithms, and BIM to create a robust data-driven framework for real-time anomaly detection and classification for maintenance decision-making. The accelerometer installed on the axle box of in-service trains collects dynamic data in the form of longitudinal (x), lateral (y), and vertical (z) accelerations. These real-time measurements are then processed using the CNN model, enabling the system to detect and classify anomalies in rail gauge geometry with high accuracy. To further improve detection and classification performance, advanced signal-processing or machine-learning techniques can be used to preprocess and extract meaningful features from the raw accelerometer data.

The integration of machine learning with BIM plays a crucial role in enhancing the visualization and interpretation of detected anomalies. By mapping the location and severity of gauge issues onto a BIM framework, the system provides a spatially contextualized view of the rail network, enabling maintenance teams to precisely identify and assess problematic sections. This spatial–temporal mapping not only improves situational awareness but also streamlines the maintenance workflow by prioritizing activities based on the urgency of detected anomalies and operational requirements. The use of BIM ensures that resources are allocated efficiently, thereby reducing downtime and optimizing overall maintenance processes.

Additionally, the system supports automated maintenance planning by feeding anomaly detection results into a decision-support mechanism. This functionality enables the prioritization of maintenance schedules, focusing on critical segments that pose the highest risks. Real-time alerts are also generated for severe anomalies, allowing immediate action to mitigate potential derailments and enhance passenger safety. The ability to address gauge issues proactively reduces the likelihood of unexpected failures and contributes to the long-term operational efficiency and reliability of the rail network.

This use case is particularly valuable in high-speed rail networks, urban metro systems, and remote or inaccessible rail segments, where safety, efficiency, and operational continuity are critical. By implementing this integrated approach, railway operators can adopt predictive maintenance strategies that align with modern asset management principles. The system’s ability to provide real-time data insights, coupled with its capacity for automated decision-making, demonstrates significant potential in transforming traditional maintenance practices into a more autonomous, cost-efficient, and safety-focused process. This study highlights the role of advanced sensing technologies, machine learning, and BIM in driving innovation in railway maintenance and underscores the importance of integrating digital solutions into critical infrastructure management.

To develop long-term maintenance strategies, the predictions derived from the developed machine-learning model can be seamlessly integrated with existing maintenance planning frameworks and systems. By doing so, railway operators and maintenance personnel will be better informed about the timing and urgency of required maintenance actions based on the detected anomalies’ severity and frequency. For example, anomalies that are detected early can be classified into categories that indicate their criticality, allowing maintenance teams to prioritize tasks efficiently. This also helps in scheduling interventions at the most opportune times, preventing unnecessary downtime and minimizing disruptions to the railway network. In addition to severity-based prioritization, integrating the model’s predictive capabilities with real-time monitoring systems offers a comprehensive approach to maintenance. By incorporating historical data, current sensor readings, and machine learning-driven predictions, maintenance intervals can be optimized, ensuring that maintenance is performed proactively before issues escalate into major failures. This approach not only extends the life of track components but also improves the overall safety and reliability of the railway system. Furthermore, the application of predictive maintenance strategies can significantly reduce maintenance costs by minimizing unplanned repairs and optimizing resource allocation. By ensuring that maintenance resources, such as personnel and equipment, are deployed when and where they are most needed, operators can make more efficient use of their budgets. Ultimately, combining these advanced data-driven approaches with traditional maintenance practices will lead to more effective, cost-efficient, and long-term strategies that improve the performance and longevity of railway infrastructure.

## 6. Conclusions

This study demonstrates the successful integration of accelerometer data, machine learning, and BIM to address the critical issue of detecting and classifying railway tight- and wide-gauge anomalies. By employing accelerometer data collected during regular train operations and processing them to train the CNN model, the proposed approach achieves high detection accuracy and reliable severity classification. The integration of BIM provides a powerful spatial and temporal framework, enabling efficient visualization, monitoring, and management of track anomalies. The combined system not only supports real-time condition monitoring but also enhances predictive maintenance practices by identifying potential anomalies early, reducing unplanned downtime, and optimizing resource allocation. The findings highlight the effectiveness of advanced sensing technologies and machine learning in improving railway safety and operational efficiency. The inclusion of BIM facilitates better asset management by storing and contextualizing data, supporting automated decision-making, and enabling a shift toward data-driven maintenance strategies. The results of this study, with detection and classification accuracies exceeding 95%, underscore the potential of this approach to transform traditional railway maintenance into a more proactive, cost-efficient, and safety-oriented process. This study not only offers an advanced tool for rail operators but also provides a framework for adopting more intelligent, proactive maintenance practices in the industry.

Despite the high detection accuracy achieved, the system’s performance may be influenced by factors such as sensor calibration, environmental conditions, and the availability of high-quality data. Additionally, the complexity of implementing BIM in existing railway infrastructures could pose challenges for full-scale adoption.

The proposed solution faces several limitations and challenges. Key limitations include reliance on high-quality, consistent data for model training, potential difficulties in generalizing the model across different railway networks, and the need for precise sensor installation and calibration. Real-time data processing and integration with existing systems may also present technical barriers, and the risk of overfitting remains a concern. Operational disruptions during sensor installation, scalability issues, and ongoing maintenance requirements could affect implementation, as well as the initial cost and resource allocation. Additionally, challenges in data interoperability and the adoption of new technologies within railway organizations may hinder smooth deployment. Addressing these limitations will require continued research, testing, and effective change management to ensure successful integration in practice.

Future research could explore the integration of additional sensor types and machine-learning models to improve detection sensitivity and generalization across diverse railway conditions. Further studies could also investigate the scalability of the proposed system across different railway networks and its adaptability to real-time operational settings. Additionally, the impact of incorporating real-time environmental data, such as weather conditions and track wear, could be studied to further enhance the system’s predictive capabilities and robustness.

These advancements provide railway operators with a robust tool for ensuring system reliability and minimizing risks, paving the way for future research and implementation in railway infrastructure management.

## Figures and Tables

**Table 1:** Comparisons between results from UM and ADAMS/Rail based on Manchester benchmark rolling stock.

Validated Parameters	Results from UM	Results from ADAMS/Rail	Difference (%)
Lateral wheelset displacement (mm)			
Wheelset 1	−6.9	−7.2	4.2%
Wheelset 2	7.6	7.2	5.6%
Wheelset 3	−6.9	−7.2	4.2%
Wheelset 4	7.4	7.2	2.8%
Longitudinal force (kN)			
Left wheel			
Wheelset 1	2.1	3.1	32.3%
Wheelset 2	−16.0	−15.7	1.9%
Wheelset 3	0.9	1.9	52.6%
Wheelset 4	−17.5	−17.5	0.0%
Right wheel			
Wheelset 1	−3.1	−3.5	11.4%
Wheelset 2	15.7	15.3	2.6%
Wheelset 3	−1.7	−2.2	22.7%
Wheelset 4	17.2	12.8	34.4%
Lateral force (kN)			
Left wheel			
Wheelset 1	32.2	31.1	3.5%
Wheelset 2	1.5	1.6	6.3%
Wheelset 3	19.9	19.0	4.7%
Wheelset 4	4.1	3.3	24.2%
Right wheel			
Wheelset 1	−23.1	−22.6	2.2%
Wheelset 2	−21.9	−21.4	2.3%
Wheelset 3	−25.1	−24.6	2.0%
Wheelset 4	−10.8	−9.5	13.7%
Vertical force (kN)			
Left wheel			
Wheelset 1	−54.4	−54.4	0.0%
Wheelset 2	−39.9	−39.6	0.8%
Wheelset 3	−49.3	−49.4	0.2%
Wheelset 4	−44.3	−44.3	0.0%
Right wheel			
Wheelset 1	−55.1	−55.0	0.2%
Wheelset 2	−68.7	−68.9	0.3%
Wheelset 3	−59.2	−59.2	0.0%
Wheelset 4	−64.9	−64.9	0.0%
Average			8.4%

**Table 2:** Data variation for MBS simulations.

List of Data Variation	Variation	Step of Variation	Unit
Types of track gauge anomalies	Tight and wide gauges	N/A	N/A
Speeds of rolling stocks	50–250	12.5	km/h
Weights of rolling stocks	15–40	2.5	Tons
Sizes of track gauge anomalies	−10 (tight gauge)–20 (wide gauge)	5	mm

**Table 3:** Response category.

Response Category	Inspection and Verification	Action
Emergency 1 (E1)	Before the next train	Before the next train
Emergency 2 (E2)	Within 2 h or the next train, whichever is later	Within 24 h
Priority 1 (P1)	Within 24 h	Within 7 days
Priority 2 (P2)	Within 7 days	Within 28 days
Priority 3 (P3)	Within 28 days	Program for maintenance
Normal (N)	N/A	Routine inspection

**Table 4:** Tight-gauge anomalies and response category.

Tight Gauge (mm)	Wide Gauge (mm)	Track Speed (Normal/Passenger) (km/h)					
		20/20	40/40	60/60	80/90	100/115	115/160
<10	<21	N	N	N	N	N	N
10	21–22	N	N	N	N	P3	P2
11–12	23–26	N	N	N	P3	P2	P1
13–14	27–28	N	N	P3	P2	P1	E2
15–16	29–30	N	P3	P2	P1	E2	E2
17	31–32	P2	P2	P1	E2	E2	E2
18	33–34	P1	P1	E2	E2	E2	E1
19–20	35–37	E2	E2	E2	E2	E1	E1
>20	>37	E1	E1	E1	E1	E1	E1

**Table 5:** List of tuned hyperparameters.

**Model**	**List of Tuned Hyperparameters**	
CNN	The number of convolutional layers The number of filters The number of kernels The number of max pooling layers Pool sizes The number of hidden layers The number of hidden nodes	Activation function Batch size Learning rate Momentum Optimizer Dropout

**Table 6:** Optimal hyperparameters from tuning.

Model	List of Tuned Hyperparameters	Optimal Parameters
CNN	The number of convolutional layers	2
	The number of filters	64 (Conv1) and 32 (Conv2)
	The number of kernels	1
	The number of max pooling layers	2 (follow the Conv layers)
	Pool sizes	2
	The number of hidden layers	2
	The number of hidden nodes	100
	Activation function	ReLU and Softmax (output layer)
	Batch size	8
	Learning rate	0.001
	Momentum	0.99
	Optimizer	Adam
	Dropout	0.25 (before the 2nd pooling layer)

**Table 7:** Results from cross-validation.

Epoch	K-Fold Cross-Validation	Accuracy of Training Data	Accuracy of Testing Data
10	1	0.99	0.98
	2	0.99	0.99
	3	1	0.99
	4	0.97	0.86
	5	0.99	0.99
20	1	1	0.99
	2	1	0.99
	3	1	0.99
	4	1	0.99
	5	1	0.99
30	1	1	0.99
	2	1	0.99
	3	1	0.99
	4	1	0.99
	5	1	0.98
40	1	1	0.99
	2	1	0.99
	3	1	0.98
	4	1	0.98
	5	1	0.99

**Table 8:** Model performance.

Class	Precision	Recall	F1-Score
0	1	1	1
1	1	1	1
2	1	1	1
3	1	1	1
4	1	0.98	0.99
5	0.98	1	0.99
6	1	1	1
		Training accuracy	1.00
		Testing accuracy	0.99

**Table 9:** Confusion matrix.

		Actual						
		Class 0	Class 1	Class 2	Class 3	Class 4	Class 5	Class 6
**Predicted**	**Class 0**	64	0	0	0	0	0	0
	**Class 1**	0	48	0	0	0	0	0
	**Class 2**	0	0	63	0	0	0	0
	**Class 3**	0	0	0	59	0	0	0
	**Class 4**	0	0	0	0	50	1	0
	**Class 5**	0	0	0	0	0	55	0
	**Class 6**	0	0	0	0	0	0	53

## Data Availability

Dataset available upon request from the authors.
